# Trimebutine prevents corneal inflammation in a rat alkali burn model

**DOI:** 10.1038/s41598-024-61112-4

**Published:** 2024-05-27

**Authors:** Hitoshi Goto, Takeshi Arima, Akira Takahashi, Yutaro Tobita, Yuji Nakano, Etsuko Toda, Akira Shimizu, Fumiki Okamoto

**Affiliations:** 1 Department of Ophthalmology, Nippon Medical School, Bunkyo-Ku, Tokyo, 113-8603 Japan; 2 Department of Analytic Human Pathology, Nippon Medical School, Bunkyo-Ku, Tokyo, 113-8603 Japan

**Keywords:** HMGB1, RAGE, Trimebutine, Cornea, Alkali burn injury, Inflammation, Molecular medicine, Eye diseases

## Abstract

Alkaline burns to the cornea lead to loss of corneal transparency, which is essential for normal vision. We used a rat corneal alkaline burn model to investigate the effect of ophthalmic trimebutine solution on healing wounds caused by alkaline burns. Trimebutine, an inhibitor of the high-mobility group box 1-receptor for advanced glycation end products, when topically applied to the burned cornea, suppressed macrophage infiltration in the early phase and neutrophil infiltration in the late phase at the wound site. It also inhibited neovascularization and myofibroblast development in the late phase. Furthermore, trimebutine effectively inhibited interleukin-1β expression in the injured cornea. It reduced scar formation by decreasing the expression of type III collagen. These findings suggest that trimebutine may represent a novel therapeutic strategy for corneal wounds, not only through its anti-inflammatory effects but also by preventing neovascularization.

## Introduction

Normal vision depends on corneal transparency. The ocular surface may suffer serious damage from alkaline burns, leading to loss of transparency^1,2^. For corneal wounds to heal properly and yield good visual outcomes, scar development and neovascularization must be suppressed.

High-mobility group box 1 (HMGB1) is a damage-associated molecular pattern involved in various physiological and pathophysiological processes such as inflammation, immune responses, and tissue healing post-damage. It signals through toll-like receptors (TLR) and advanced glycation end products (receptor for advanced glycation end products, RAGE)^3,4^. HMGB1 can also act as a pro-inflammatory cytokine to directly or indirectly activate a complex inflammatory cascade, prompting the rapid migration of inflammatory cells to wounded tissues. HMGB1 is released into the extracellular environment passively by dead cells or actively by inflammatory cells^5^. It also contributes to angiogenesis and fibrosis associated with inflammation^6^. Recent studies have highlighted that HMGB1 is a potential therapeutic target for various eye diseases^7^.

Several drugs have been found to inhibit the interaction between HMGB1 and RAGE and suppress inflammatory effects. Papaverine, a vasodilator and antispasmodic agent, was recently discovered to bind to the extracellular domain of RAGE and inhibit the interaction between HMGB1 and RAGE, thereby diminishing the HMGB1-initiated inflammatory response^8^. Glycyrrhizin, found in the crude drug licorice, binds to HMGB1 and inhibits HMGB1-mediated inflammation after corneal injury^6,9^. Trimebutine, a papaverine mimetic, has emerged as a more potent inhibitor of the HMGB1–RAGE signaling pathway than papaverine^10^. Due to its antimuscarinic and μ-opioid agonist actions, trimebutine is used as an antispasmodic drug to treat irritable bowel syndrome and reduce gastrointestinal motility disorders, regulating bowel and colon movements and relieving abdominal pain^11,12^.

However, to the best of our knowledge, no ophthalmological studies have focused on the effects of trimebutine. In this study, we evaluated the anti-inflammatory, anti-angiogenic, and anti-fibrotic effects of trimebutine in a rat model of corneal alkaline burns.

## Results

### Effect of trimebutine on corneal wound healing after alkaline burn

In this study, a rat model of corneal alkaline trauma was developed. The rats were divided into a trimebutine eye drop group (n = 8) and a vehicle eye drop group (n = 8), and eye drops were administered twice daily starting immediately after injury.

**Figure 1:**
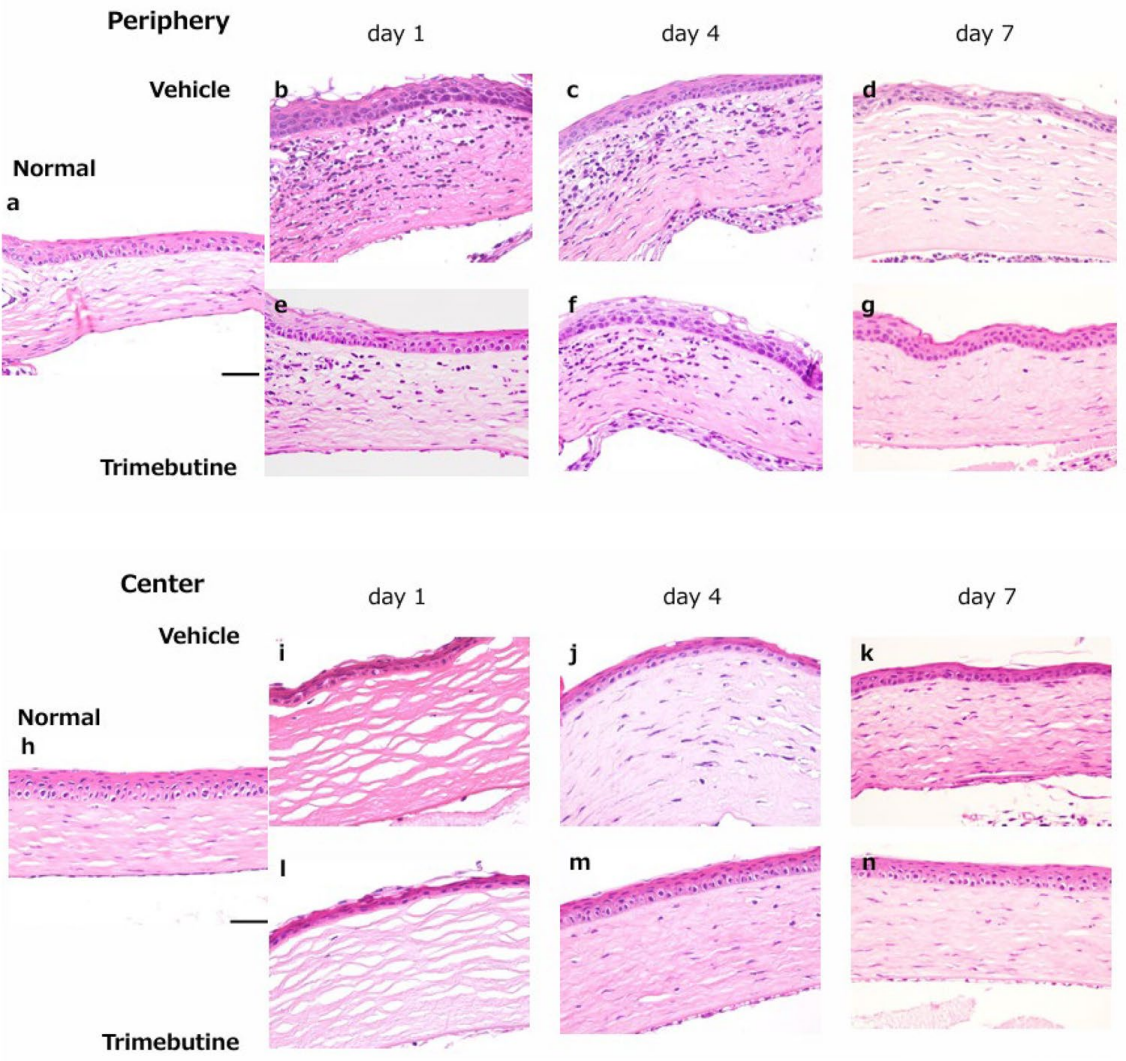

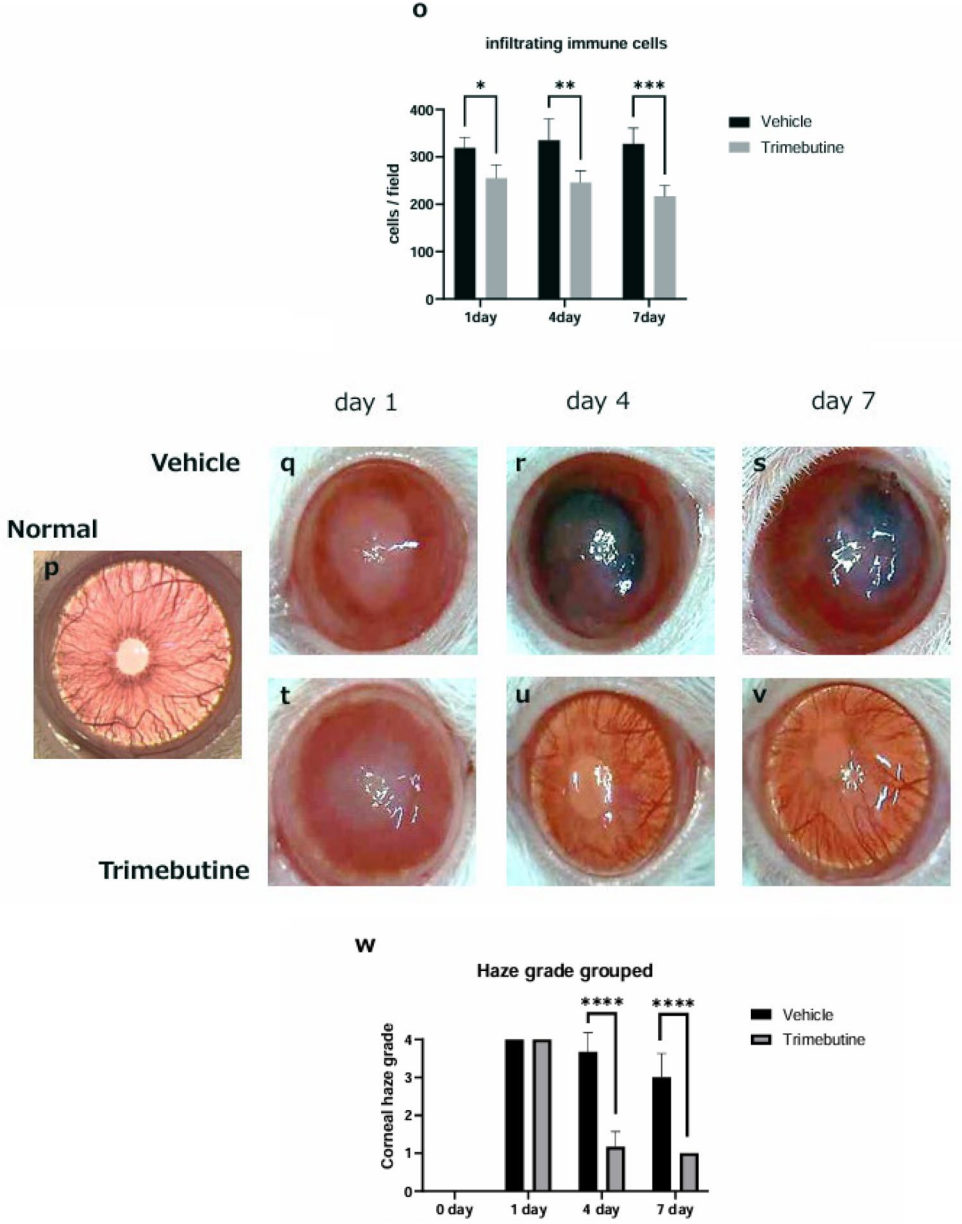
Wound healing process after alkaline burns. Time-dependent changes in the normal group (**a**: peripheral region, **h**: central region) and vehicle group (**b**–**d**: peripheral region, **i**–**k**: central region) and trimebutine group (**e**–**g**: peripheral region, **l**–**n**: central region). Bar, 50 μm. By days 4 and 7, the inflammatory cell infiltrates had migrated to the central corneal area. Compared with the vehicle-treated group (**j**, **k**), the trimebutine-treated group (**m**, **n**) showed less inflammatory cell infiltration (**o**). On day 1, unlike in normal eyes, the blood vessels in the iris appeared to have vanished on the macroscopic images, and the corneal epithelial defect became immediately visible as a circle (**p**, **q**, **t**). After day 4, in the vehicle group, the cornea completely lost transparency, and bleeding was observed in the anterior chamber (**r**, **s**). However, in the trimebutine instillation groups, corneal stromal opacity and neovascularization were less pronounced. Blood vessels in the iris could also be identified (**u**, **v**). Change in corneal haze grading at each endpoint for the trimebutine and vehicle groups (**w**). Data are presented as the mean ± standard deviation (n = 8 samples/group) *P < 0.05, **P < 0.01, ***P < 0.001, ****P < 0.0001.

Hematoxylin–eosin (HE) staining was performed to compare the corneal wound healing process between the vehicle- and trimebutine-treated groups (Fig. 1 a-n). On day 1, after the alkali burn, infiltration due to various inflammatory cells was increased in the corneal periphery (Fig. 1 b, e). Stromal edema was observed in the central cornea (Fig. 1 i, l). By days 4 and 7, the inflammatory cells had migrated to the central corneal area. Compared with the vehicle-treated group (Fig. 1 j, k), the trimebutine-treated group showed decreased inflammatory cell infiltration (Fig. 1 m, n). Throughout the study period, trimebutine suppressed the number of inflammatory cell infiltrates (Fig. 1 o).

On day 1, unlike in normal eyes, the blood vessels in the iris appeared to have vanished on the macroscopic images, and the corneal epithelial defect became immediately visible as a circle (Fig. 1 p, q, t). After day 4, in the vehicle group, the cornea completely lost transparency, and bleeding was observed in the anterior chamber (Fig. 1 r, s). However, there was less corneal stromal opacity and fewer neovascular vessels in the trimebutine group. Blood vessels in the iris could also be identified (Fig. 1 u, v). Moreover, the haze grading system previously reported by Fantes et al.^13^ was used to grade corneal haze.: grade 0, totally clear cornea; grade 1, a more pronounced haze that does not obscure fine iris details; grade 2, mild obscuration of iris details; grade 3, moderate obscuration of iris details and of the lens; grade 4, severe opacification of the stroma with no visibility of iris details. Three ophthalmologists used blind observation to determine the haze grade (Fig. 1 w).

### HMGB1 and RAGE are upregulated during corneal wound healing, and trimebutine effectively attenuates their release

**Figure 2:**
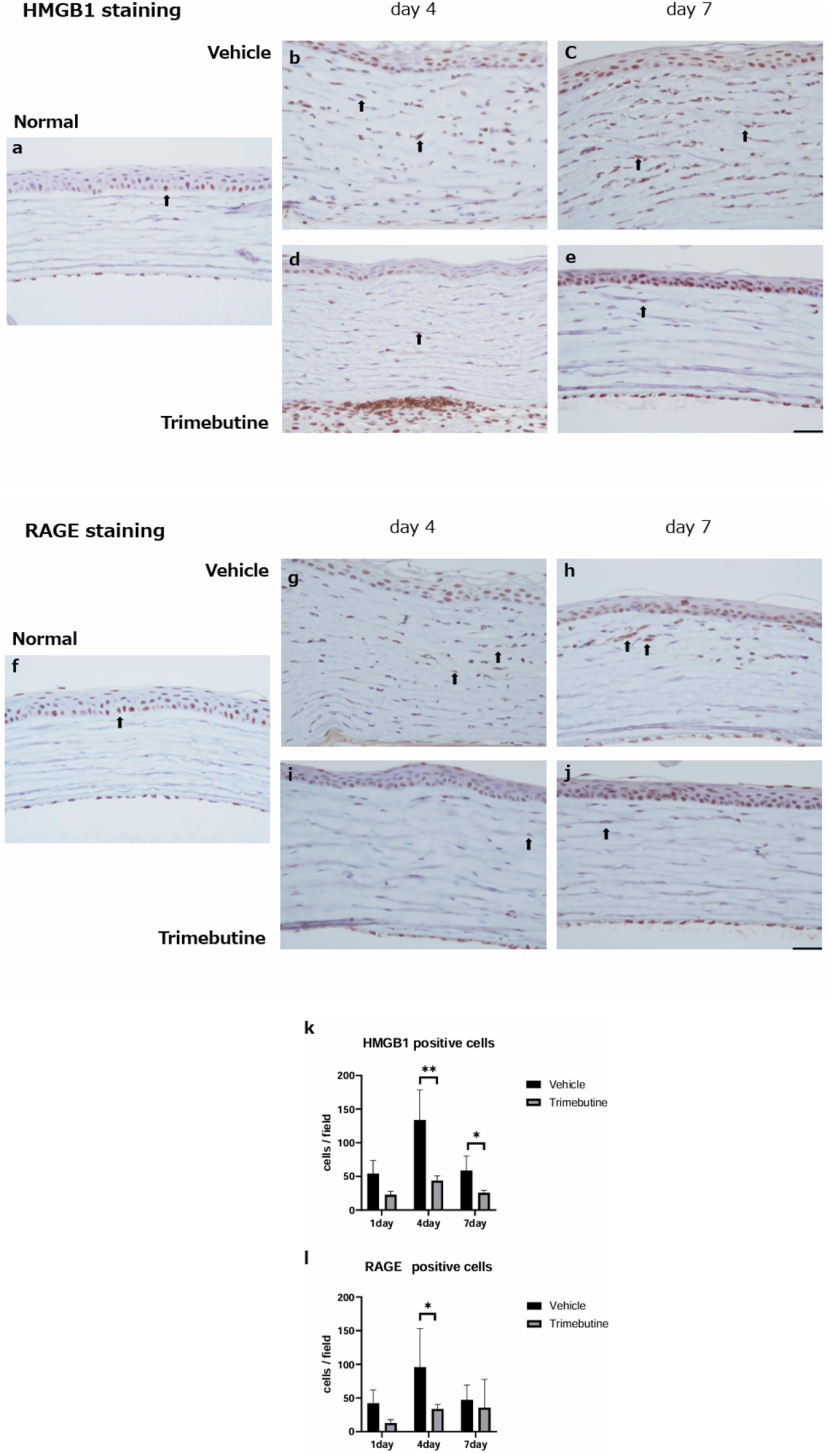
HMGB1 staining and RAGE staining. In the normal cornea, HMGB1 and RAGE are expressed in the epithelium (**a**, **f**). Bar, 50 μm. Alkaline injury resulted in a broader distribution of HMGB1 and RAGE in the corneal stromal layer than in uninjured normal corneas, suggesting infiltration of inflammatory cells expressing these molecules in the corneal stroma (**b**, **c**, **g**, **h**). Trimebutine reduced the number of positively stained cells for HMGB1 and RAGE in the corneal stroma (**d**, **e**, **i**, **j**). Compared with the vehicle group, especially on day 4, trimebutine effectively decreased the number of HMGB1 and RAGE positive cells (k, l). Data are presented as the mean ± standard deviation (n = 8 samples/group) *P < 0.05, **P < 0.01. HMGB1, high mobility group box 1; RAGE, receptor for advanced glycation end products.

Next, we evaluated HMGB1 and RAGE expression in normal corneas and during corneal wound healing to determine whether HMGB1 and RAGE are potent modulators of corneal epithelial healing events involved in the inflammatory cascade. HMGB1 and RAGE were expressed in the epithelium (Fig. 2 a, f). Alkaline injury resulted in a broader distribution of HMGB1 and RAGE in the corneal stromal layer than in the uninjured normal corneas, suggesting the infiltration of inflammatory cells expressing these molecules into the corneal stroma (Fig. 2 b, c, g, h). Trimebutine reduced the number of cells positively stained for HMGB1 and RAGE in the corneal stroma (Fig. 2 d, e, i, j). Enumeration of the number of HMGB1- and RAGE-positive cells showed that the number of positive cells peaked on day 4 in the vehicle group (Fig. 2 k, l). Compared to the vehicle group, especially on day 4, trimebutine effectively decreased the number of HMGB1- and RAGE-positive cells (Fig. 2 k, l).

### Anti-inflammatory effects of the trimebutine ophthalmic solution

**Figure 3:**
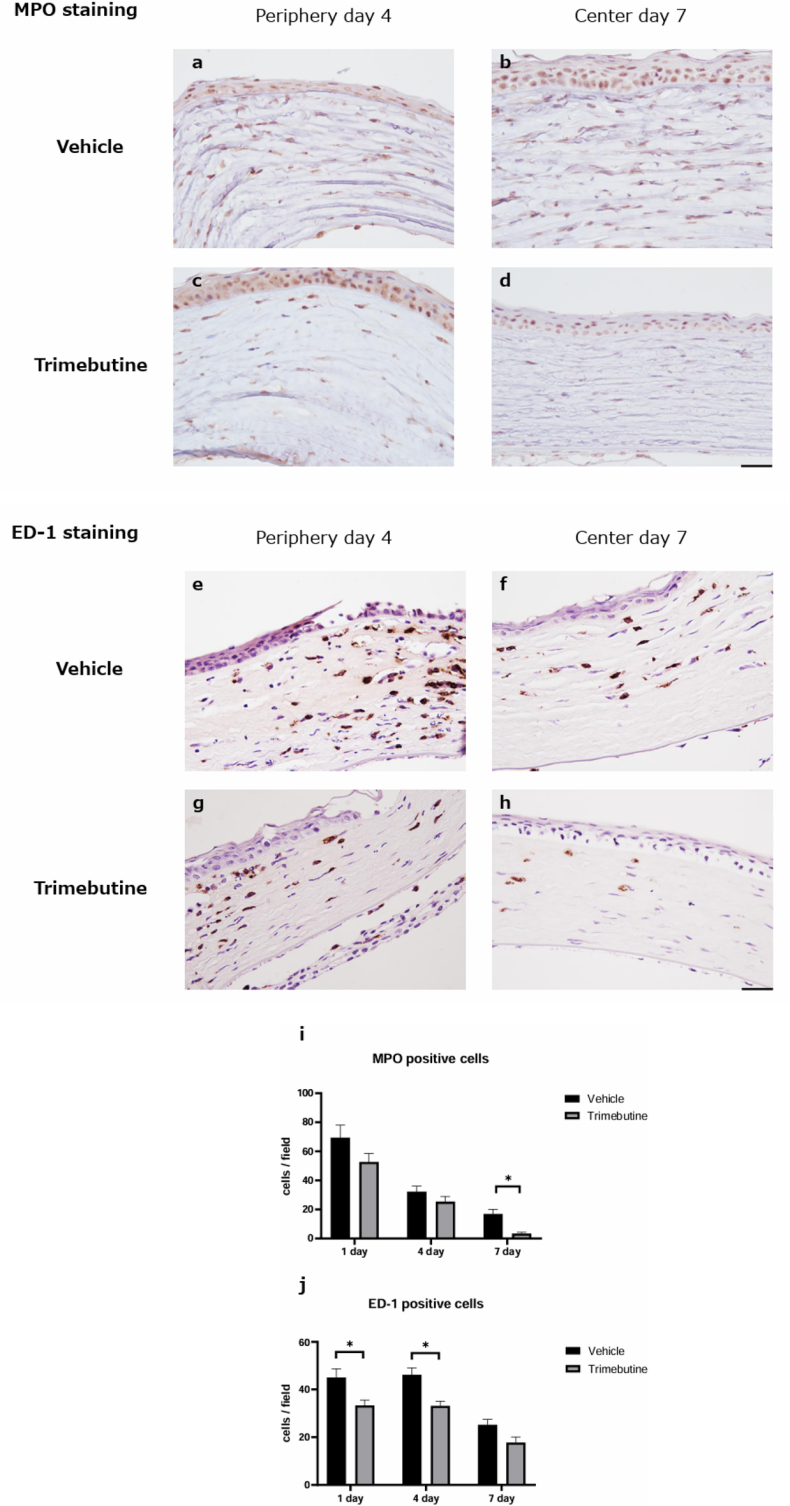
MPO staining. To investigate the infiltrating neutrophils, myeloperoxidase (MPO) staining was performed (**a**–**d**). Bar, 50 μm. The trimebutine ophthalmic solution significantly decreased the degree of infiltrating neutrophils compared with vehicle by day 7 (**i**). Data are presented as the mean ± standard deviation (n = 8 samples/group). *P  < 0.05. ED-1 staining. To investigate the infiltrating macrophages, CD68 antibody (ED-1) staining was performed (**e**–**h**). Bar, 50 μm. Trimebutine treatment significantly suppressed ED-1-positive macrophages on days 1 and 4 compared with vehicle treatment (**j**). Data are presented as the mean ± standard deviation (n = 8 samples/group) *P  < 0.05.

To investigate neutrophil infiltration, myeloperoxidase (MPO) staining was performed (Fig. 3 a-d). On day 1, the number of neutrophils peaked in the injured corneas in both groups. Trimebutine ophthalmic solution significantly decreased the number of infiltrating neutrophils compared with the vehicle by day 7 (Fig. 3 i). We also investigated macrophage infiltration through immunohistochemical analysis with a CD68 antibody (ED-1) (Fig. 3 e–h). On day 4, the number of ED-1-positive macrophages peaked in the injured corneas in both groups. Trimebutine treatment significantly suppressed ED-1-positive macrophages on days 1 and 4 compared with vehicle treatment (Fig. 3 j).

### Suppression of neovascularization by trimebutine

**Figure 4:**
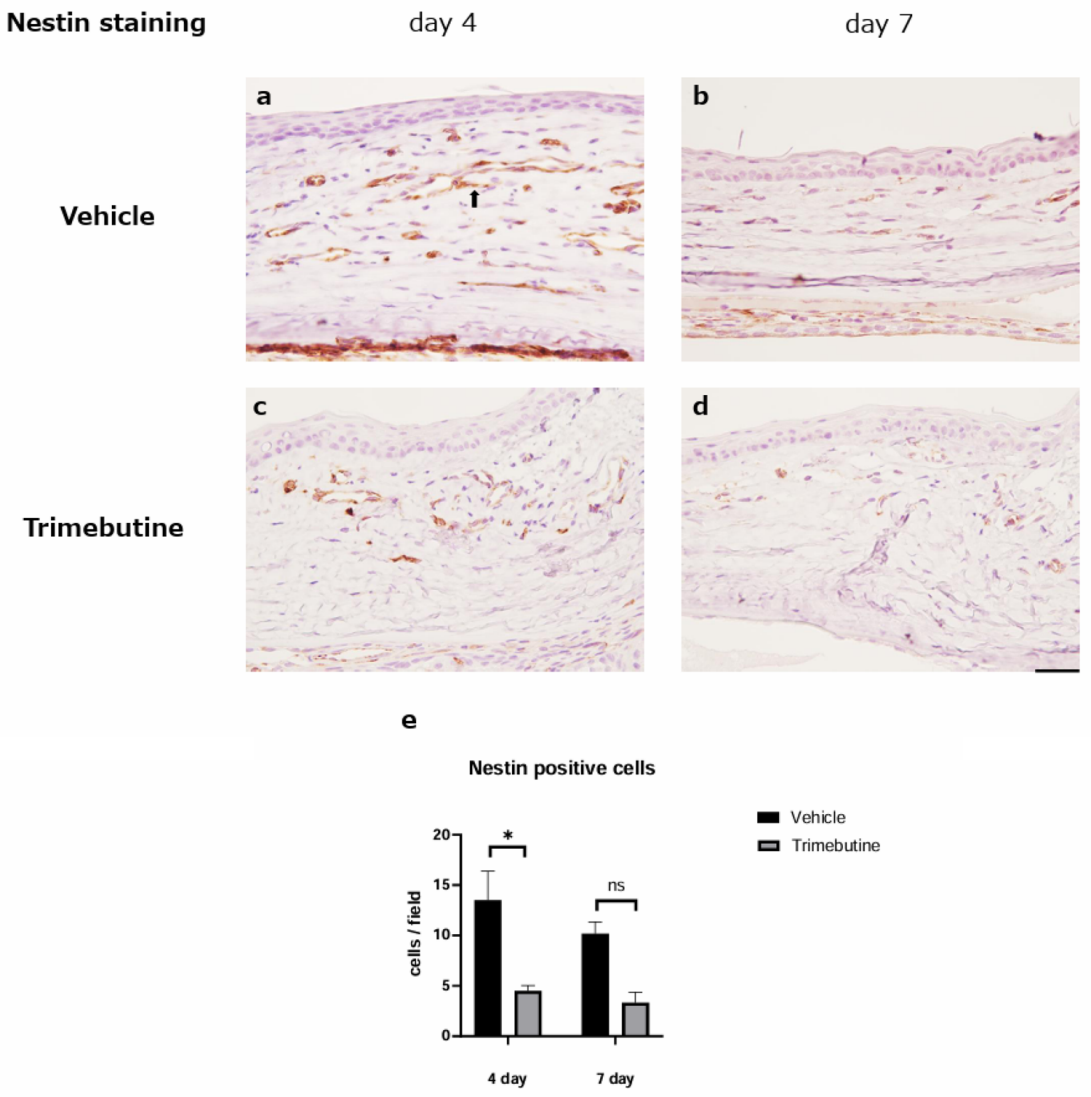
Nestin staining. Immunohistochemical analysis of nestin in the vehicle (**a**–**b**) and trimebutine (**c**–**d**) groups. Bar, 50 μm. The black arrow indicates nestin-positive endothelial cells of a new blood vessel (**a**). Nestin-positive newly formed endothelial cells were observed in the corneal limbus from day 4 after the alkali burn (**a**, **c**). In the early phase (day 4), newly formed blood vessels were significantly fewer in the trimebutine groups than in the vehicle group (**e**). Data are presented as the mean ± standard error (n = 8 samples/group). *p < 0.05.

Immunohistochemical analysis of nestin expression was performed to investigate neovascularization. Nestin stains the immature vascular endothelial cells associated with angiogenesis. Newly formed nestin-positive endothelial cells were observed in the corneal limbus from day 4 after the alkali burn (Fig. 4 a, c). On days 4 and 7, fewer nestin-positive endothelial cells were observed in the trimebutine group than in the vehicle group. A statistically significant difference was observed on day 4 (Fig. 4 a-b, c-d, e). These results indicated that the ophthalmic solution of trimebutine prevented the development and migration of neovascularization.

### Anti-fibrotic effects of the trimebutine ophthalmic solution

**Figure 5:**
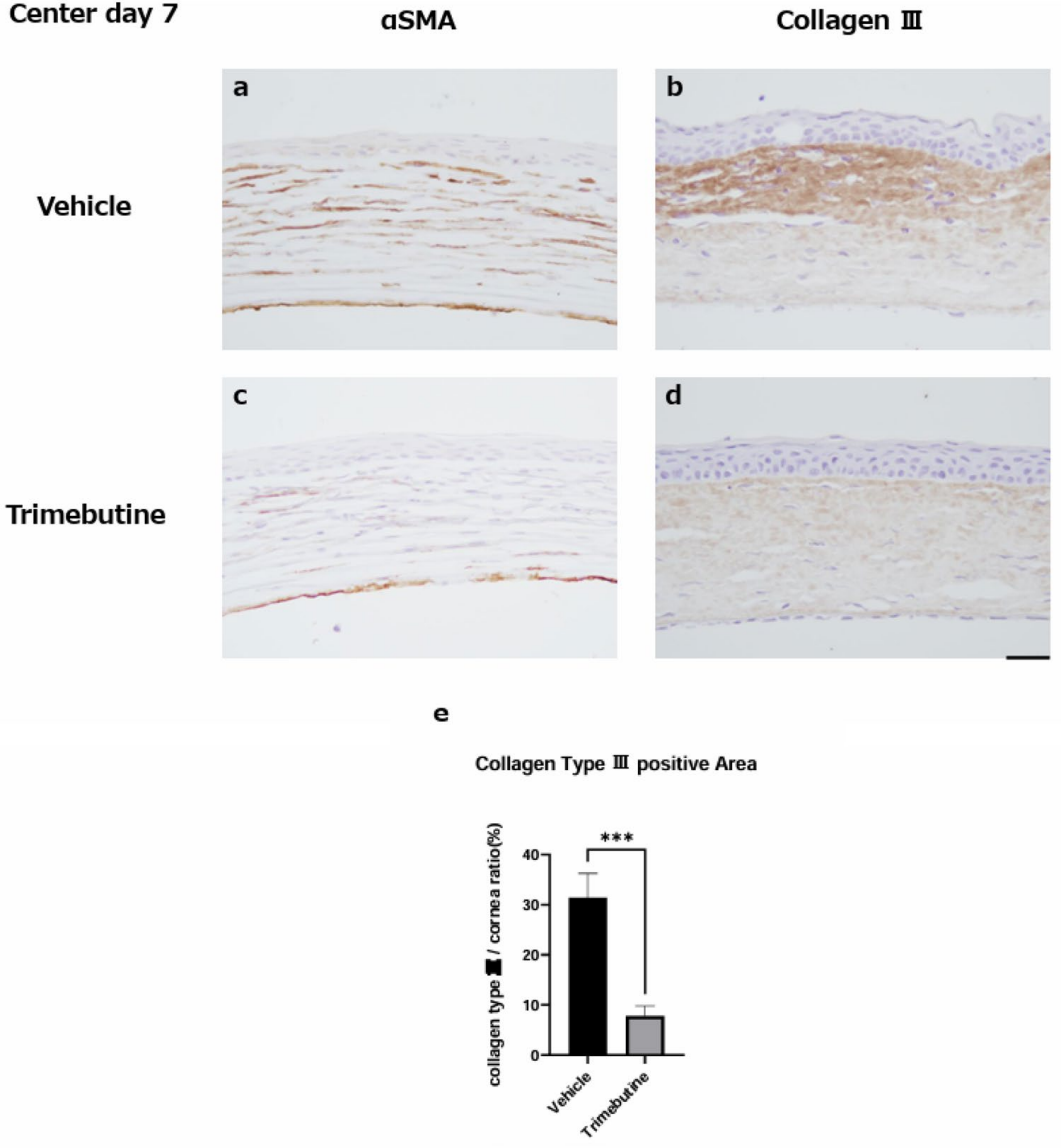
Regeneration of the corneal stroma. Although type III collagen and alpha-smooth muscle actin (α-SMA) were observed at the injured area during the healing process, the trimebutine group (**c**, **d**) exhibited lower expression of type III collagen and α-SMA than the vehicle group (**a**, **b**). Bar, 50 μm. Bar chart of percentages of the expression rate of type III collagen within the corneal stroma during the late phase showing that the trimebutine group had significantly lower percentages of type III collagen than the vehicle group (**e**). Data are presented as the mean ± standard error (n = 8 samples/group). ***P  < 0.001.

To examine fibrotic alterations in the corneal stroma during wound healing, we focused on alpha-smooth muscle actin (α-SMA)-positive myofibroblasts and type III collagen. On day 7 post-injury, accumulation of α-SMA-positive myofibroblasts and type III collagen was detected (Fig. 5 a, b). However, the trimebutine group showed a decreased buildup of α-SMA and type III collagen (Fig. 5 c, d). When the trimebutine and vehicle groups were compared, the stained area of type III collagen in the ocular area was significantly reduced in the trimebutine group (Fig. 5 e).

### Inflammatory cytokines

Real-time reverse transcription polymerase chain reaction (RT-PCR) was performed to compare the mRNA expression levels of inflammatory cytokines in corneas, including tumor necrosis factor (TNF)-α, IL-6, interleukin (IL)-1β, and chemokine (C–C motif) ligand 2 (CCL2), before the alkaline injury and 4 and 7 days after.

**Figure 6:**
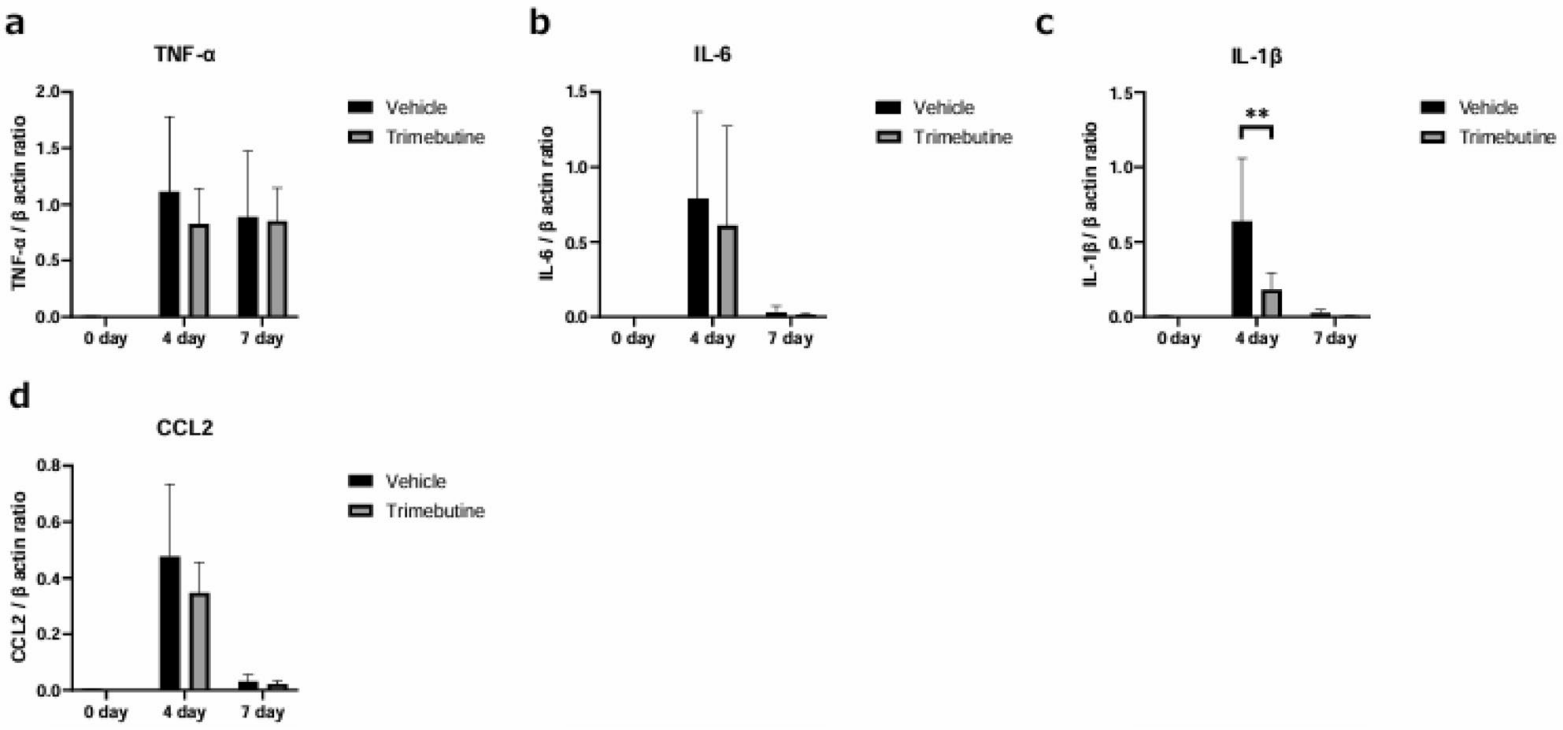
Expression of proinflammatory cytokine mRNAs in the cornea before alkaline injury and 4 and 7 days after. The mRNA expression levels of tumor necrosis factor (TNF)-α (**a**), interleukin (IL)-6 (**b**), IL-1β (**c**), and chemokine (C–C motif) ligand 2 (CCL2) (**d**) were measured. The level of each cytokine peaked on day 4 as did HMGB1- and RAGE-positive cells. IL-6, IL-1b, and CCL2 levels decreased on day 7. Only IL-1β levels decreased with trimebutine treatment (**a**–**d**). Data are expressed as the mean ± standard error (n = 8 samples/group). **P  < 0.01. HMGB1, high mobility group box 1; RAGE, receptor for advanced glycation end products.

The mRNA expression level of each cytokine peaked on day 4, which coincided with the peak in HMGB1- and RAGE-positive cells detected in the cornea (Fig. 2 k, l). The mRNA expressions of IL-6, IL-1b, and CCL2 decreased on day 7. Among these cytokines, only IL-1β was reduced by trimebutine treatment (Fig. 6 a-d).

### Effects of the trimebutine ophthalmic solution on the normal cornea

**Figure 7:**
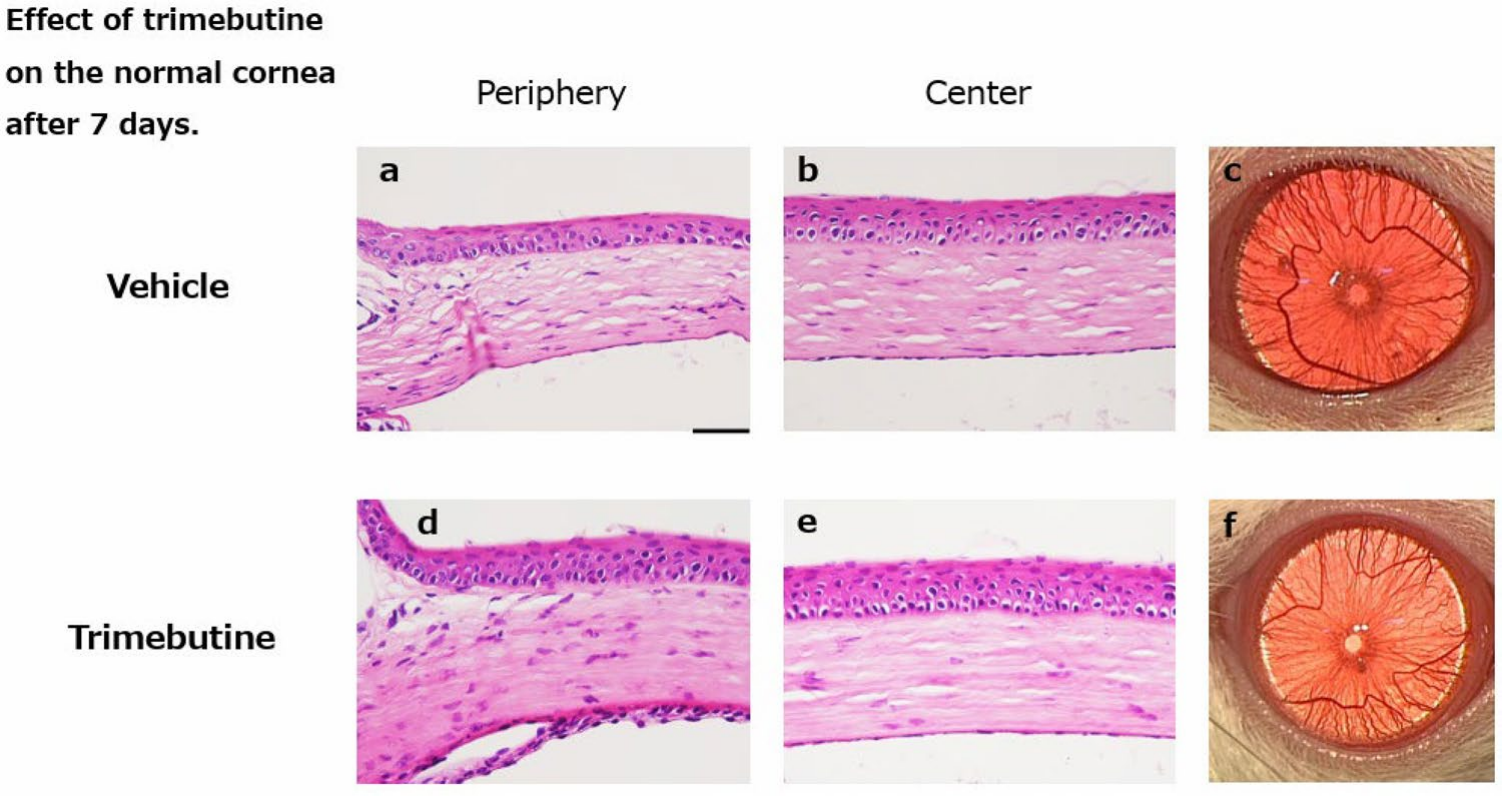
Effect of trimebutine on the normal cornea after 7 days. HE staining of the vehicle group (**a**: peripheral region, **b**: central region) and the trimebutine group (**d**: peripheral region, **e**: central region). Bar, 50 μm. There were no notable differences on the HE staining or macroscopic images (**c**, **f**) between the two groups. HE, hematoxylin and eosin.

The ocular surface toxicity of the trimebutine ophthalmic solution on the normal cornea was also investigated. Trimebutine or vehicle was administered twice daily to normal corneas, and the rats were sacrificed after 7 days. Histological analysis demonstrated no apparent difference between the two groups (Fig. 7 a-f).

## Discussion

We aimed to demonstrate the effect of inhibiting the HMGB1-RAGE signaling pathway using a rat corneal alkali burn model, which is widely used to study treatment mechanisms, acute inflammation, and neovascularization in injured corneas. Trimebutine, which is used as an antispasmodic agent, can inhibit the HMGB1-RAGE signaling pathway and be used as an eye drop solution according to the findings of this study.

HMGB1 and its ligands contribute to the progression of multiple ocular diseases, including wound healing^7^. HMGB1 has been implicated in infectious keratitis^14^, chronic autoimmune uveitis^15^, acute glaucoma^16^, diabetic retinopathy^17^, and alkali-induced corneal neovascularization^18^. In this study, in the trimebutine-treated group, trimebutine reduced inflammatory cell infiltration, decreased the levels of HMGB1 and RAGE, prevented neovascularization and migration, and exhibited anti-fibrotic effects.

HMGB1 and RAGE are expressed in the corneal epithelium of normal rat corneas. Upon injury, both are secreted into the stroma by epithelial and inflammatory cells^9,19^. In this study, the expression of both decreased in the trimebutine-treated group. Glycyrrhizin, an HMGB1-RAGE inhibitor, reduces neutrophil infiltration into the corneal stroma and promotes corneal wound healing^9^. Neutrophils not only remove the cellular components released from dead cells but also cause uncontrolled chronic inflammation^20^. HMGB1 induces neutrophil migration to injured tissues and is believed to activate an amplification loop during neutrophil-mediated injury^5^. The trimebutine-treated group exhibited reduced neutrophil infiltration of the corneal stroma. The expression of HMGB1 and RAGE peaked on day 4, with a significant difference in cell counts between the two groups. This result was thought to be the cause of the difference in neutrophil counts between the two groups on day 7. The fact that the RAGE-positive cell count did not differ significantly on day 7 may reflect cells other than neutrophils.

Previous reports have suggested that HMGB1-RAGE directly links necrotic cells to neutrophils without requiring macrophage-mediated signaling^5^. Our findings indicated that trimebutine also reduced macrophage infiltration, suggesting that HMGB1-RAGE inhibition regulates macrophage infiltration in injured corneas. The time course of infiltration of neutrophils and macrophages differed, with the peak for macrophages observed from days 1 to 4 and that for neutrophils observed on day 1. Because these are also different from the time course of HMGB1 and RAGE expression, both of which peaked on day 4, cells expressing HMGB1 and RAGE should be carefully evaluated and identified.

HMGB1 exacerbates inflammation by inducing cytokine and chemokine release and inflammatory cell infiltration^21^. It promotes monocyte mobilization by activating the RAGE/nuclear factor (NF)-κB signaling pathway and inhibits monocyte apoptosis^22^. HMGB1 also acts as a mediator to release TNF, IL-1β, IL-6, and other cytokines from both mobilized leukocytes and resident immune cells^23^. In a mouse model of Kawasaki disease, among a number of cytokines, only IL-1β was suppressed, and the disease condition improved^24^. Similarly, in the present study, IL-1β regulation seems to be associated with beneficial therapeutic outcomes in corneal wound healing.

Neovascularization after corneal injury can be caused by various factors, such as inflammation and hypoxia^25,26^. A recent study showed that administration of glycyrrhizin, an HMGB1-RAGE inhibitor, decreased VEGF and CD31 expression and ultimately suppressed corneal neovascularization^6^. In the present study, trimebutine treatment reduced the proliferation of immature vascular endothelial cells during the early phase and suppressed neovascularization during the late phase.

The normal cornea consists of regulated type I collagen that maintains transparency by helping refract light^27^. Although type III collagen and myofibroblasts play an important role in wound healing, irrelevant deposition of collagen can result in an opaque cornea^28^, frequently observed after alkali burns. Our findings indicate that α-SMA-positive myofibroblasts and type III collagen were accumulated in stromal areas, and suppressed by trimebutine, leading to less scar formation.

This study has some limitations. Pathological analysis revealed the therapeutic effect of trimebutine on corneal injury; however, the underlying molecular mechanism is not fully understood. In this study, the therapeutic effect of trimebutine was evident, and its impact on temporal changes in inflammatory cells and mediators and on subsequent scar formation was demonstrated. However, the molecular mechanisms by which the complex HMGB1-RAGE pathway is affected by trimebutine treatment and the cell types that express HMGB1 and RAGE have not been studied in detail. A multifaceted evaluation that includes signaling molecules such as NF-κB and MAP kinase ERK1/2, which are reportedly inhibited by trimebutine, is required in the future^10^.

The endpoints for this experiment were 1, 4, and 7 days. The HMGB1 and RAGE cascades are considered late mediators of the inflammatory response and are induced 24–36 h post-injury^9^. Although the endpoints of this experiment were reasonable for assessing neovascularization and fibrosis, a shorter interval between the initial endpoints may be more rigorous for a detailed assessment of the initial process and the elucidation of mechanisms in the future. In addition, in this study, we performed a macroscopic assessment of corneal stromal opacity. Quantitative evaluation using, for example, anterior segment optical coherence tomography would be desirable in the future.

In conclusion, the findings of this study suggest that trimebutine administration can inhibit inflammation, fibrosis, and neovascularization in corneas with alkali burn. Currently, steroid eye drops are commonly used for their anti-inflammatory effect in clinical practice. However, some patients experience difficulties with long-term use of steroid eye drops owing to side effects, such as increased intraocular pressure^29^. Future studies are required to compare the efficacy of trimebutine with steroid eye drops and determine if trimebutine can be used as an additional treatment to reduce reliance on steroid eye drops. In summary, this study demonstrated that trimebutine, an existing medication, has the potential to be a novel treatment for severe corneal wounds characterized by inflammation, neovascularization, and scar formation.

## Materials and methods

### Animals and ethics statement

The study was carried out in compliance with the ARRIVE guidelines. Eight-week-old male *Wistar* rats (*n* = 8 per group/endpoint; Sankyo Laboratory Service, Tokyo, Japan) were used in all experiments. All animal experiments were performed in accordance with the Association for Research in Vision and Ophthalmology Statement for the Use of Animals in Ophthalmic and Vision Research. The Experimental Animal Ethics Review Committee of Nippon Medical School, Tokyo, approved the current research procedures (approval number: 2020–099, December 5, 2020).

### Experimental procedures

Under general isoflurane anesthesia, a circular filter paper 3.2 mm in diameter was immersed in 1 N NaOH and placed on the central cornea of each rat for 1 min to create a corneal alkali burn. After alkali exposure, the ocular surface was washed with 40 mL physiological saline solution. The trimebutine ophthalmic solution or vehicle solution was administered twice daily to the alkali-burned corneas. The vehicle solution was filter-purified 100 mL NaCl-based phosphate buffered saline (0.01 M; pH 7.4), which was prepared with 232 g disodium hydrogen-phosphate 12-water, 23.7 g sodium dihydrogen phosphate dihydrate, 4000 mL distilled water, and 0.1 mL polyoxyethylene sorbitan monooleate (Wako Pure Chemical Industries, Osaka, Japan). The 0.2% trimebutine ophthalmic solution was prepared by adding 0.2 g of 2-(dimethylamino)-2-phenylbutyl 3,4,5-trimethoxybenzoate maleate (Tokyo Chemical Industry Co., Ltd.) to 100 mL of the vehicle solution. At each endpoint (1, 4, and 7 days after the alkali burn), the rats were sacrificed by exsanguination under isoflurane anesthesia. Pathological and molecular biological evaluations were performed on the enucleated eyes.

### Histological and immunohistochemical analysis

At 1, 4, and 7 days after the alkali burn, the eyes were enucleated for histological and immunohistochemical analyses (n = 8 per time point), fixed in 10% buffered formalin, and embedded in paraffin for light microscopic analysis. Thin sections were prepared with a microtome. The sections were made at the corneal apex and were 2.5 μm thick. Deparaffinized tissues were stained with HE for histopathological examination. MPO staining was performed to detect infiltrating neutrophils. Primary antibodies used for immunohistochemical analysis included polyclonal rabbit anti-HMGB1 (GeneTex Inc, North America), polyclonal rabbit anti-RAGE (Bioss Inc, Boston), polyclonal mouse anti-MPO (*Arigo Biolaboratories Corp*, *Taiwan*), monoclonal mouse anti-rat ED-1 (BMA, Nagoya, Japan; to detect infiltrating macrophages), monoclonal mouse anti-nestin (Nestin; Merck Millipore, Darmstadt, Germany; to detect endothelial cells of immature blood vessels), and goat anti-type III collagen (Southern Biotech, Birmingham, AL, USA). Paraffin-embedded tissues fixed in 10% buffered formalin were used for immunohistochemical analysis. Tissues were stained using the standard avidin–biotin-peroxidase complex technique. For the selection of tissue sections of the cornea, first, the entire cornea was observed at a 40 × field of view to determine the most central section, which was observed in a 400 × field of view, and both sides of the section were selected in a 400 × field of view. A total of three fields of view were used to observe the central section. For observation of the periphery, each corneal limbus was selected in a 400 × field of view. These selections were combined for a total of five fields of view. The average of the five fields of view of the cornea at 400 × magnification was calculated as the cell count. The number of cells that were immunohistochemically positive was counted by one researcher. If the cells formed intercellular junctions, nuclei were identified and counted as one. The corneal area at 400 × magnification in three fields of view of the central cornea was measured using ImageJ (National Institutes of Health, Bethesda, MD, USA)^30–33^.

### Real-time RT-PCR

To examine the mRNA expression levels of TNF-α, IL-6, IL-1β, and CCL2, we used real-time RT-PCR (n = 8 per time point). Total RNA was obtained from the corneas using ISOGEN II (Nippon Gene, Tokyo, Japan) following the manufacturer’s protocol. The RNA content and purity (A260/A280) were measured using a NanoDrop ND-1000 V3.2.1 spectrophotometer from NanoDrop Technologies in Wilmington, DE, USA. cDNA libraries were synthesized from the whole RNA using a High Capacity cDNA Reverse Transcription kit (Thermo Fisher Scientific) following the manufacturer’s protocol. Genes of interest were amplified (2 min at 50 °C, 10 min at 95 °C, and 45 cycles of denaturation at 95 °C for 15 s and annealing at 60 °C for 60 s) using the QuantStudioTM 3 Real-Time PCR System (Thermo Fisher Scientific), THUNDERBIRD SYBR qPCR Mix (TOYOBO, Osaka, Japan), and particular primers. Normalized mRNA expression values were determined by calculating the relative quantity of the housekeeping gene, β-actin. PCR primers used in this study are listed in Table 1.

**Table 1:** PCR primers used in this study.

Gene	Forward primer sequence (5′–3′)	Reverse primer sequence (5′–3′)
β-actin	GCAGGAGTACGATGAGTCCG	ACGCAGCTCGTAACAGTCC
TNF-α	AAATGGGCTCCCTCTCATCAGTTC	TCTGCTTGGTGGTTTGCTACGAC
IL-6	GTCAACTCCATCTGCCCTTCAG	GGCAGTGGCTGTCAACAACAT
IL-1β	TACCTATGTCTTGCCCGTGGAG	ATCATCCCACGAGTCACAGAGG
CCL2	CCAATGAGTAGGCTGGAGAGC	ACCCATTCCTTCTTGGGGTC

### Statistical analyses

All results are expressed as the mean ± standard error. Different groups were compared using one-way analysis of variance followed by the Tukey–Kramer post-hoc test. Statistical significance was set at p < 0.05. All analyses were performed using GraphPad Prism software (version 10.0.2; GraphPad Software, San Diego, CA, USA).

## Conclusions

Our findings indicate that trimebutine may represent a novel therapeutic strategy for corneal wounds, not only through its anti-inflammatory effects but also by preventing neovascularization.

## Data Availability

All data generated or analyzed during this study are included in this published article.
